# Distribution of Peripheral PrP*^Sc^* in Sheep with Naturally Acquired Scrapie

**DOI:** 10.1371/journal.pone.0097768

**Published:** 2014-05-14

**Authors:** María Carmen Garza, Marta Monzón, Belén Marín, Juan José Badiola, Eva Monleón

**Affiliations:** 1 Centro de Encefalopatías Espongiformes y Enfermedades Emergentes, Universidad de Zaragoza, Zaragoza, Spain; 2 Centre for Prions and Protein Folding Diseases, University of Alberta, Alberta, Canada; 3 Departamento de Anatomía e Histología Humanas, Universidad de Zaragoza, Zaragoza, Spain; Creighton University, United States of America

## Abstract

Accumulation of prion protein (PrPSc) in the central nervous system is the hallmark of transmissible spongiform encephalopathies. However, in some of these diseases such as scrapie or chronic wasting disease, the PrPSc can also accumulate in other tissues, particularly in the lymphoreticular system. In recent years, PrPSc in organs other than nervous and lymphoid have been described, suggesting that distribution of this protein in affected individuals may be much larger than previously thought. In the present study, 11 non-nervous/non-lymphoid organs from 16 naturally scrapie infected sheep in advanced stages of the disease were examined for the presence of PrPSc. Fourteen infected sheep were of the ARQ/ARQ PRNP genotype and 2 of the VRQ/VRQ, where the letters A, R, Q, and V represent the codes for amino-acids alanine, arginine, glutamine and valine, respectively. Adrenal gland, pancreas, heart, skin, urinary bladder and mammary gland were positive for PrPSc by immunohistochemistry and IDEXX HerdChek scrapie/BSE Antigen EIA Test in at least one animal. Lung, liver, kidney and skeletal muscle exhibited PrPSc deposits by immunohistochemistry only. To our knowledge, this is the first report regarding the presence of PrPSc in the heart, pancreas and urinary bladder in naturally acquired scrapie infections. In some other organs examined, in which PrPSc had been previously detected, PrPSc immunolabeling was observed to be associated with new structures within those organs. The results of the present study illustrate a wide dissemination of PrPSc in both ARQ/ARQ and VRQ/VRQ infected sheep, even when the involvement of the lymphoreticular system is scarce or absent, thus highlighting the role of the peripheral nervous system in the spread of PrPSc.

## Introduction

Transmissible spongiform encephalopathies (TSEs; also known as prion diseases) are a group of neurodegenerative diseases that affects humans and animals. The main feature of these diseases is the deposition of a misfolded form of the prion protein (hereafter referred to as PrP^Sc^) in the central nervous system (CNS). In some TSEs, including scrapie in sheep and goats, chronic wasting disease (CWD) in deer and variant Creutzfeldt-Jakob disease (vCJD) in humans, PrP^Sc^ has been detected in diverse tissues and organs outside the CNS [1], [2].

Scrapie was the first TSE to be identified and is considered to be the archetype of this group of diseases. In naturally acquired scrapie, evidence suggests that the main route of entry of the agent is through the gut-associated lymphoid tissues, which then spreads to the CNS by centripetal transport along the peripheral nervous system (PNS; [3], [4]). However, the scrapie agent may also propagate to other organs; PrP^Sc^ deposits in natural scrapie infections have been detected in placenta [5], skeletal muscle [6], kidney [7], [8], skin [9] and liver [10]. In addition, PrP^Sc^ has also been associated with inflammation foci in different organs such as the lung and mammary glands from scrapie infected sheep [11], [12], [13].

Tissue distribution of the scrapie agent may be influenced by several different factors including host genetics, the dose of the infectious agent and the strain of the agent [1], [14], [15]. The ovine *PRNP* genotype determines susceptibility to scrapie and is one of the best studied factors known to affect the spread of the scrapie agent. The ancestral *PRNP* gene encodes for the amino-acids alanine (A), arginine (R) and glutamine (Q) at codons 136, 154 and 171 (hereafter referred to as haplotype ARQ) and it is the most common haplotype found in Spanish ovine breeds [16]. Polymorphisms at codons 136 (substitution of A by valine (V); haplotype VRQ) and 171 (substitution of Q by R; haplotype ARR) are associated with an increased susceptibility or resistance to sheep scrapie, respectively. The majority of studies addressing the spread of infection through the body in naturally acquired scrapie use sheep carrying the most susceptible genotype (VRQ/VRQ), which are associated with wide PrP^Sc^ dissemination throughout the sheep [17]. In ARQ/ARQ scrapie infected sheep, the degree of the systemic spread of infection is less clear. It appears that ARQ/ARQ and VRQ/ARQ sheep develop natural scrapie with longer incubation periods and less dissemination of PrP^Sc^ than VRQ/VRQ sheep [18], [19], [20]. Here, we examined the main characteristics and tissue distribution of PrP^Sc^ in organs outside the CNS and LRS from scrapie infected ARQ/ARQ sheep. We used naturally infected animals and conventional methods for PrP^Sc^ detection (i.e., immunohistochemistry) that reflect the pathophysiology and transmission of the disease better than other experimental models or ultrasensitive PrP^Sc^ detection techniques [1], [21].

## Materials and Methods

### Animals

The study population included 16 scrapie infected sheep from 7 scrapie outbreaks detected within the framework of the Spanish TSE surveillance program. Animals were selected upon exhibition of clinical signs compatible with scrapie [22], and infection was confirmed post-mortem by PrP^Sc^ immunodetection in the brainstem at the level of the obex (hereafter referred to as brainstem). With two exceptions, all animals displayed the ARQ/ARQ genotype and were of the Rasa Aragonesa breed or a cross breed.

Ten control sheep were selected from flocks in which no scrapie cases have been reported to date, and these sheep died of different causes other than scrapie. Animals were confirmed to be negative for scrapie by using immunohistochemistry (IHC) to detect the presence of PrP^Sc^ in lymphoid tissue and the CNS. Individual details of sheep used in this study are provided in Tables 1 (scrapie infected sheep) and S1 (control sheep).

**Table 1:** Details of the scrapie infected sheep used in the study.

Animal No.	Age^1^ (years)	Outbreak	Genotype	PrP^Sc^ in brainstem		PrP^Sc^ in spleen	
				IHC^2^	EIA^3^	IHC^2^	EIA^3^
1	4	A	VRQ/VRQ	+++	1∶4096	+++	1∶64
2	3	B	VRQ/VRQ	+++	1∶4096	++	1∶16
3	4	C	ARQ/ARQ	+++	1∶16384	++	1∶64
4	4	C	ARQ/ARQ	+++	1∶16384	++	1∶64
5	2	D	ARQ/ARQ	+++	1∶16384	++	1∶64
6	>6	D	ARQ/ARQ	+++	1∶4096	−	−
7	5	E	ARQ/ARQ	+++	1∶16384	−	−
8	>6	E	ARQ/ARQ	+++	1∶4096	++	1∶64
9	4	E	ARQ/ARQ	+++	1∶4096	+++	1∶64
10	5	E	ARQ/ARQ	+++	1∶4096	++	1∶64
11	4	E	ARQ/ARQ	+++	1∶16384	+++	1∶64
12	5	E	ARQ/ARQ	+++	1∶4096	++	1∶64
13	5	F	ARQ/ARQ	+++	1∶4096	+++	1∶256
14	4	F	ARQ/ARQ	+++	1∶4096	+++	1∶64
15	5	F	ARQ/ARQ	+++	1∶4096	++	1∶16
16	4	G	ARQ/ARQ	+++	1∶4096	+++	1∶64

From each animal the identification number, age, flock of origin (outbreak), genotype and immunohistochemical and IDEXX EIA test results from brainstem and spleen are included.

1 Age was determined by the dental status of the animals.

2 PrP^Sc^ presence and score: positive (+) or negative (−) for IHC.

3 Limit detection of PrP^Sc^ by IDEXX EIA test.

This study was approved by the Ethics Committee for Animal Experiments of the University of Zaragoza (Permit Number: PI02/08) and was carried out in strict accordance with the recommendations for the care and use of experimental animals and in agreement with the national legislation (R.D. 1201/2005).

### Tissue Collection

Infected animals were euthanized by intravenous injections of sodium pentobarbital and exsanguination, and the necropsy was performed immediately. The following organs were collected from each sheep: brain, palatine tonsils, spleen, ileum, heart, lung, pancreas, liver, adrenal gland, kidney, urinary bladder, ovary, uterus, skin and skeletal muscle. Except for palatine tonsils and ileum, all tissues were divided into two halves. One half was formalin-fixed and paraffin-embedded for further histopathological analysis, and the other half was frozen and stored at −80°C until used for the IDEXX HerdChek scrapie/BSE Antigen EIA Test. Palatine tonsils and ileum (Peyer’s patch) were examined only by IHC.

### Immunohistochemistry

Formalin-fixed tissues were trimmed and processed according to standard histopathological procedures. A 4 µm tissue section from each sample was stained with haematoxylin and eosin for microscopic examination.

For PrP^Sc^ detection by IHC, 3 serial tissue sections (4 µm) spaced 200 µm apart were obtained and assessed as previously described [23]. Briefly, sections were pre-treated with 98% formic acid, proteinase K (4 µg/ml; F. Hoffmann La Roche Ltd, 211 Switzerland) and hydrated autoclaving. Then, tissue sections were incubated with a blocking reagent and immunolabeled using an automated immunostainer. Sections were incubated with an L42 primary antibody (0.046 µg/ml; R-Biopharm, Darmstadt, Germany) at room temperature for 30 minutes. EnVision was used as a visualisation system and diaminobenzidine as the chromogen. Finally, sections were counterstained with haematoxylin. If necessary, PrP^Sc^ immunostaining with antibody L42 was confirmed by immunostaining with the monoclonal antibody F89 (0.5 µg/ml; NC-Neopharma) using the protocol describe above. Unless stated otherwise, all products were obtained from Dako (Denmark A/S, Denmark).

The presence/absence of PrP^Sc^ deposits and their characteristics were assessed in 3 tissue sections except for the CNS and LRS samples in which only 1 section was examined. For brainstem and spleen samples, PrP^Sc^ signal was subjectively scored based on the extent of immunostaining [24], [25]. In the brainstem, + staining was characterised as the accumulation of PrP^Sc^ in the dorsal motor nucleus of the vagus nerve, ++ staining was characterised as the accumulation of PrP^Sc^ in the dorsal motor nucleus of the vagus nerve and the adjacent nuclei, and +++ staining was characterised as widespread PrP^Sc^ throughout the entire section. In the spleen, + staining was characterised as <10% of the lymphoid follicles containing PrP^Sc^ deposits, ++ staining was defined as 10 to 50% of the lymphoid follicles containing PrP^Sc^ deposits, and +++ staining was defined as >50% of the lymphoid follicles containing PrP^Sc^ deposits.

### IDEXX HerdChek Scrapie/BSE Antigen EIA Test

All samples were analysed with the ‘IDEXX HerdChek scrapie/BSE Antigen EIA Test’ (hereafter identified as the IDEXX EIA) according to the manufacturer’s instructions with a previous pre-homogenization step included. Briefly, 2 grams of each frozen sample were cut into small pieces with two scalpels until the tissue appeared homogeneous. Three hundred mg of the sample were placed into tissue-disruption tubes containing ceramics beads and buffer, and were subjected to 3 cycles of homogenization in the TeSeE Precess 48 Homogenizer (Bio-Rad). Each cycle consisted of 2 agitation phases of 45 seconds at 6500 rpm with 60 seconds of waiting time between phases. The samples were allowed to cool for 5 minutes between cycles. One hundred and twenty µl of the resulting homogenate was mixed with 30 µl of the diluent in the working plate. One hundred µl of the diluted sample were transfer to the antigen capture plate provided. The plate was agitated for 1 hour at room temperature and washed 6 times with wash solution 1. A conditioning buffer was subsequently added to each well and incubated for 10 minutes at room temperature, and the plate was washed again for 3 more cycles. Immobilised PrP^Sc^ was detected by incubating the plate with a peroxidase conjugated anti-PrP antibody and was visualised with TMB substrate. After incubating for 15 minutes, the reaction was stopped by the addition of 1 M hydrochloric acid, and the absorbance was read at both 450 nm and 620 nm.

To compare the amount of PrP^Sc^ present among all animals, a detection limit was determined for the brainstem and spleen samples. Brainstem and spleen homogenised samples were diluted in the buffer present in the tissue-disruption tubes (from the kit) to obtain the following progressive dilutions: 1∶4, 1∶16, 1∶64, 1∶256, 1∶1024, 1∶4096, 1∶16384 and 1∶65416. The dilutions were examined by the IDEXX EIA according to the manufacturer’s instructions as previously described. The detection limit was determined to be the last dilution at which these tissue samples tested positive.

In the case of the 10 control sheep, only the brainstem, spleen, liver, pancreas and urinary bladder samples were examined by IDEXX EIA.

## Results

Scrapie infection was demonstrated via the immunodetection of PrP^Sc^ in the brainstem of 16 clinically infected sheep. By IHC, all brainstem samples were scored as +++, and the results of the IDEXX EIA displayed optical density (OD) values ranging from 3.438 to 4.065. The dilution series reached a detection limit between 1∶4096 and 1∶16384. Of the 16 sheep that exhibited PrP^Sc^ accumulation in the brainstem, 14 were also positive for PrP^Sc^ in all lymphoid tissue samples examined (palatine tonsils, ileum Peyeŕs Patches and spleen) and 2 were negative in all lymphoid samples (No. 6, 7; Table 1). Positive spleen samples were further studied: all samples exhibited ++ or +++ staining scores by IHC and IDEXX EIA values ranged from 1.866 to 3.637 (except for No. 15, OD 0.752). In the spleen samples, the dilution series reached a detection limit between 1∶16 and 1∶256. The Individual details of PrP^Sc^ detection by IHC and the detection limits measured by the IDEXX EIA in the brainstem and spleen from the scrapie infected sheep are provided in Table 1.

With regards to the other tissues, PrP^Sc^ was detected in all organs examined except for the ovary and uterus. Adrenal gland, pancreas, heart, skin, urinary bladder and mammary glands were all positive by both techniques in at least one animal. Lung, liver, kidney and skeletal muscle exhibited PrP^Sc^ deposits only by IHC, although some OD values were close to the cut-off point set for the IDEXX EIA. It should be noted that the cut-off point is determined by the software kit and has been established specifically for nervous and lymphoid tissues. In the assays performed, the cut-off point ranged from 0.163 to 0.235.

PrP^Sc^ was not detected in samples obtained from the negative-control sheep by either technique. Individual details of PrP^Sc^ detection by IHC and IDEXX EIA in each organ examined are provided in Tables 2 (scrapie infected sheep) and S2 (control sheep).

**Table 2:** Individual sheep results of PrP^Sc^ detection by IHC/IDEXX EIA in each peripheral organ studied.

Animal No.																
	1	2	3	4	5	6	7	8	9	10	11	12	13	14	15	16
**Adrenal gl.**	**+**/**+**	**+**/**+**	**+**/**+**	**+**/**+**	**+**/**−**	**+**/**−**	**+**/**−**	**+**/**+**	**+**/**+**	**+**/**+**	**+**/**+**	**+**/**−**	**+**/**+**	**+**/**+**	**+**/**+**	**+**/**+**
	2.34	0.72	0.33	2.99	0.12	0.03	0.03	2.84	3.36	2.93	3.07	0.09	3.50	1.56	3.54	0.59
**Heart**	**−**/**−**	**−**/**−**	**−**/**−**	**−**/**−**	**−**/**+**	**−**/**+**	**+**/**−**	**+**/**−**	**+**/**−**	**−**/**−**	**+**/**+**	NT	**−**/**−**	**+**/**−**	**−**/**+**	**−**/**−**
	0.03	0.03	0.04	0.05	0.24	0.93	0.12	0.05	0.03	0.06	0.43	NT	0.18	0.02	0.22	0.02
**Tongue**	**−**/**−**	**+**/**−**	NT	NT	**−**/**−**	**−**/**−**	**+**/**−**	**−**/**−**	**−**/**−**	**−**/**−**	**+**/**−**	**−**/**−**	**−**/**−**	**−**/**−**	**−**/**−**	**−**/**−**
	0.05	0.06	NT	NT	0.04	0.03	0.04	0.03	0.05	0.04	0.2	0.09	0.03	0.05	0.04	0.02
**Sk.** **Muscle**	**+**/**−**	**+**/**−**	**+**/**−**	**+**/**−**	**−**/**−**	**−**/**−**	**+**/**−**	**+**/**−**	**+**/**−**	**−**/**−**	**−**/**−**	**−**/**−**	**+**/**−**	**−**/**−**	**−**/**−**	**−**/**−**
	0.03	0.04	0.03	0.03	0.04	0.03	0.06	0.09	0.04	0.04	0.03	0.04	0.04	0.03	0.03	0.03
**Pancreas**	**−**/**−**	**−**/**−**	**−**/**−**	**+**/**+**	**−**/**−**	**−**/**−**	**−**/**−**	**+**/**+**	**−**/**−**	**−**/**−**	**−**/**−**	**−**/**−**	**+**/**−**	**−**/**−**	**−**/**−**	**−**/**−**
	0.04	0.07	0.14	1.43	0.05	0.05	0.05	0.23	0.06	0.05	0.05	0.04	0.10	NT	0.04	0.03
**Skin**	**−**/**−**	**+**/**−**	**−**/**−**	**+**/**−**	**−**/**−**	**−**/**−**	**+**/**−**	**−**/**−**	**−**/**−**	**+**/**+**	**−**/**−**	**−**/**−**	**+**/**−**	**−**/**−**	**−**/**−**	**−**/**−**
	0.12	0.04	0.07	0.07	0.05	0.05	0.05	0.04	0.06	2.56	0.07	0.04	0.05	0.04	0.05	0.06
**Urinary** **bl.**	**−**/**−**	**−**/**−**	**−**/**−**	**−**/**−**	**−**/**−**	**−**/**−**	**−**/**−**	**+**/**+**	**−**/**−**	**−**/**−**	**−**/**−**	**−**/**−**	**−**/**−**	**+**/**−**	**−**/**−**	**−**/**−**
	0.1	0.05	0.07	0.06	0.05	0.05	0.05	0.26	0.05	0.07	0.05	0.06	0.04	0.16	0.05	0.04
**Kidney**	**−**/**−**	**+**/**−**	**−**/**−**	**+**/**−**	**+**/**−**	**−**/**−**	**−**/**−**	**−**/**+**	**−**/**−**	**+**/**−**	**+**/**−**	**−**/**−**	**−**/**−**	**−**/**−**	**−**/**−**	**−**/**−**
	0.1	0.18	0.11	0.1	0.21	0.09	0.06	0.59	0.08	0.07	0.08	0.10	0.11	0.08	0.08	0.04
**Mamm.** **gl.**	**−**/**−**	NT	**+**/**−**	**−**/**−**	**−**/**−**	**−**/**−**	**+**/**−**	NC/**−**	**+**/**+**	NC/**+**	**−**/**−**	**+**/**+**	**−**/**−**	NC/**−**	**−**/**−**	**−**/**−**
	0.04	NT	0.09	0.05	0.06	0.08	0.07	0.10	0.68	0.3	0.05	0.29	0.04	0.18	0.08	0.05
**Lung**	**−**/**−**	**−**/**−**	**−**/**−**	**−**/**−**	**−**/**−**	**−**/**−**	**−**/**−**	**+**/**−**	**−**/**−**	**−**/**−**	**−**/**−**	**+**/**−**	**+**/**−**	**+**/**−**	NC/**−**	**−**/**−**
	0.05	0.07	0.07	0.06	0.05	0.06	0.06	0.08	0.07	0.07	0.05	0.05	0.05	0.04	0.05	0.03
**Liver**	**−**/**−**	NC/**−**	**−**/**−**	**−**/**−**	**−**/**−**	**−**/**−**	**−**/**−**	**+**/**−**	**−**/**−**	**+**/**−**	**+**/**−**	**−**/**−**	**−**/**−**	**−**/**−**	**−**/**−**	**−**/**−**
	0.06	0.1	0.05	0.05	0.05	0.06	0.05	0.09	0.05	0.08	0.07	0.1	0.04	0.08	0.04	0.02

The OD value obtained by IDEXX EIA is indicated for each sample.

NT, no tested. NC, no conclusive results.

### Detection of PrP^sc^ in the Adrenal Glands

**Figure 1:**
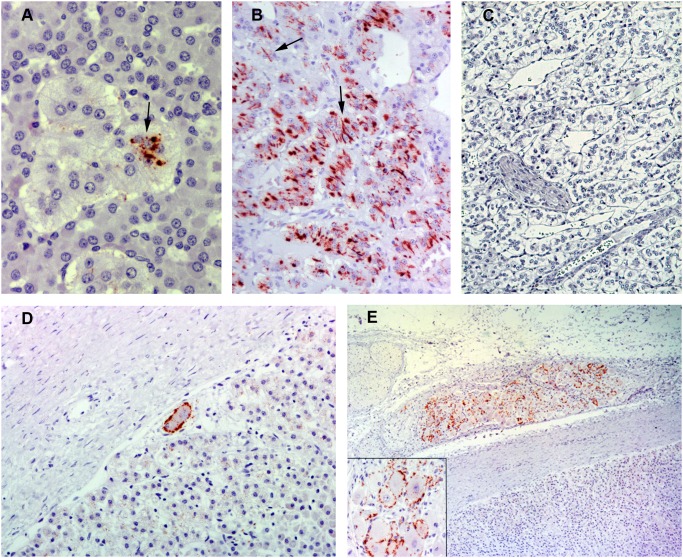
Immunohistochemical detection of PrP^Sc^ in adrenal glands using L42 PrP antibody. A) Adrenal medulla from a naturally scrapie-infected sheep (x40). Granular intracytoplasmic PrP^Sc^ immunolabeling is associated with chromaffin cells (arrow). B) Adrenal medulla from a naturally scrapie-infected sheep with an intense immunoreactivity (x20). Linear PrP^Sc^ immunolabeling can be observed also associated with chromaffin cells (arrows). C) Adrenal medulla from an uninfected control sheep in which no PrP^Sc^ immunolabeling is present (x10). D) Adrenal capsule and cortex from a naturally scrapie-infected sheep (x20). Perineuronal PrP^Sc^ immunolabeling is associated with neurons located beneath the capsule. E) Suprarenal ganglion from a naturally scrapie-infected sheep with abundant granular immunoreactivity for PrP^Sc^ (x5). A detailed image of the ganglion cells is shown in the insert picture in which PrP^Sc^ immunolabeling is observed mainly around ganglion cells.

Of the 16 animals that showed PrP^Sc^ in the CNS, 12 sheep were positive for PrP^Sc^ in the adrenal glands based on the 2 assays used (Table 2). By IHC, all animals exhibited PrP^Sc^ accumulation in the adrenal glands, mostly in the medulla. Granular intracytoplasmic labeling of chromaffin cells was observed in all cases (Fig. 1A). In medullas with widespread PrP^Sc^ accumulation, larger PrP^Sc^ deposits of irregular shape and short thick linear deposits were found also associated with chromaffin cells (Fig. 1B). No PrP^Sc^ immunolabelling was observed within medulla of adrenal glands of uninfected control sheep (Fig. 1C). In addition to the medulla, PrP^Sc^ was detected just beneath the capsule of the adrenal gland. In this case, immunolabeling in the perineuronal region of the ganglion cells (Fig. 1D) and a linear pattern thought to be associated with nerve fibres was observed. The suprarenal ganglion that surrounds the adrenal gland was positive when present in the histological sample (Fig. 1E). Although intraneuronal labeling was found in some ganglion cells, PrP^Sc^ was mainly observed in the perineuronal region related to satellite cells or neuronal processes. By IDEXX EIA testing, 12 of the 16 IHC-positive adrenal glands exhibited OD values above the cut-off point, displaying the highest values in comparison with all of the other peripheral organs. In 8 cases, OD values were included in the same range as the values obtained in spleen samples.

### Detection of PrP^sc^ in the Heart

Although only one infected sheep contained a positive heart sample via both techniques (No. 11), 4 additional animals were positive by IHC alone (No. 7, 8, 9, 14) and 3 additional animals were positive via IDEXX EIA alone (No. 5, 6, 15; Table 2). One of the IHC negative samples showed the highest OD value (No. 6; OD 0.93).

**Figure 2:**
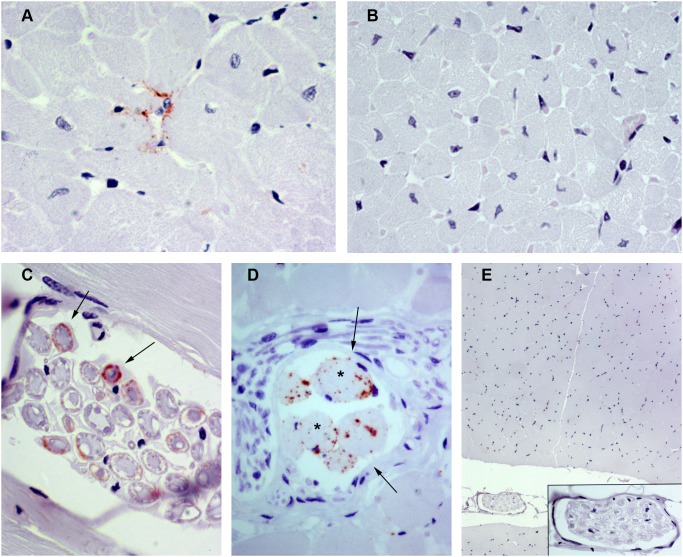
Immunohistochemical detection of PrP^Sc^ in heart and skeletal muscle using L42 PrP antibody. A) Heart from a naturally scrapie-infected sheep. PrP^Sc^ immunolabeling is located among myocardial myocytes (x63). B) Heart from an uninfected control sheep in which no PrP^Sc^ immunolabeling is present (x40). C) Cross section of a nerve fiber bundle located within a skeletal muscle sample from a naturally scrapie-infected sheep (x63). A periaxonal PrP^Sc^ immunolabeling can be observed in some nerve fibers (arrows). D) Skeletal muscle from a naturally scrapie-infected sheep in which PrP^Sc^ immunolabeling is associated with a neuromuscular spindle (cross section; x63). The neuromuscular spindle was identified by its typical structure: small groups of specialized striated muscle fibers (asterisks), which are thinner than regular muscle fibers, surrounded by a capsule of connective tissue (arows). E) Skeletal muscle from an uninfected control sheep in which no PrP^Sc^ immunolabeling is present (x10). A detailed image of a nerve fiber bundle immersed in the muscle is shown in the insert picture.

With the exception of sheep No. 8, which displayed an extensive area of immunolabeling, small amounts of PrP^Sc^ were detected by IHC. In all cases, immunolabeling was located between myocardial cells (Fig. 2A) and was often observed around blood vessel endothelia. In sheep No. 8, Congo Red staining was performed to detect amyloid deposits; however, a negative result was obtained. In sheep No. 9, a markedly positive immunostaining was also found in the epicardium and was likely associated with vasculonervous elements. No PrP^Sc^ immunolabelling was observed in heart of uninfected control sheep (Fig. 2B).

### Detection of PrP^sc^ in the Skeletal Muscle

By IHC, 3 sheep exhibited positive immunostaining of skeletal muscle samples from the tongue (No. 2, 7, 11), and the other muscles obtained from 8 sheep were also positive (No. 1–4, 7–9, 13; Table 2). With one exception (No. 8), immunolabeling was always associated with a few small nerves inside the skeletal muscle (1 or 2 positive nerve fibers per section). In transversal sections, periaxonal immunolabeling could be observed in some nerve fibres (Fig. 2C). In the case of sheep No. 8, PrP^Sc^ immunolabeling was observed within the neuromuscular spindles, as previously reported [6]. Neuromuscular spindles are highly innervated structures that consist of small groups of specialized striated muscle fibers (intrafusal fibers) surrounded by a capsule of connective tissue. In the positive neuromuscular spindles observed in the present study we detected PrP^Sc^ imunolabeling within intrafusal fibers and around them (Fig. 2D); this peri-fiber immunolabeling could be associated with the sensory nerve fibers that are spirally arranged around the mid region of intrafusal cells. No PrP^Sc^ immunolabelling was observed in skeletal muscle of uninfected control sheep (Fig. 2E). By IDEXX EIA, all of the skeletal muscle samples exhibited OD values below the cut-off, although one sample obtained from the tongue displayed an elevated OD value (No. 11; 0.2).

### Detection of PrP^sc^ in the Pancreas

**Figure 3:**
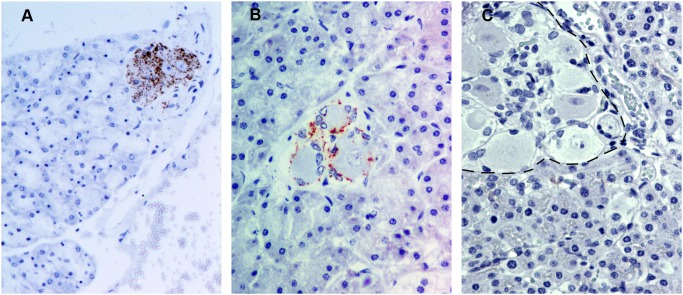
Immunohistochemical detection of PrP^Sc^ in pancreas using L42 PrP antibody. A–B) Pancreas from naturally scrapie-infected sheep. PrP^Sc^ immunolabeling was detected only in relation to intrapancreatic ganglia neurons, showing a granular intracytoplasmic (A; x20) or a perineuronal (B; x40) labelling. C) Pancreas from an uninfected control sheep in which an intrapancreatic ganglion is indicated by the dotted line (x40). No PrP^Sc^ immunolabeling is present.

In 2 sheep, PrP^Sc^ deposits were detected in the pancreas by IHC and IDEXX EIA (No. 4, 8; Table 2). By IHC, 3 pancreatic tissue samples exhibited PrP^Sc^ immunolabeling associated with structures of the PNS (No. 4, 8, 13). In these samples, intracytoplasmic and perineuronal immunolabeling was observed in the parasympathetic postganglionic neurons immersed in the pancreatic tissue (Fig. 3A, B). Occasionally the positive neurons were located in areas adjacent to the islet of Langerhans. A linear immunolabeling pattern in the connective tissue septa that separates pancreatic lobules was also observed. No PrP^Sc^ immunolabelling was observed in pancreas of uninfected control sheep (Fig. 3C). One of the 2 samples that tested positive by both techniques gave a high OD value by IDEXX EIA (No. 4; OD 1.43). Two other pancreatic samples exhibited OD values close to the cut-off point (No. 3, 13), one of which was the remaining IHC-positive sample.

### Detection of PrP^sc^ in the Skin

**Figure 4:**
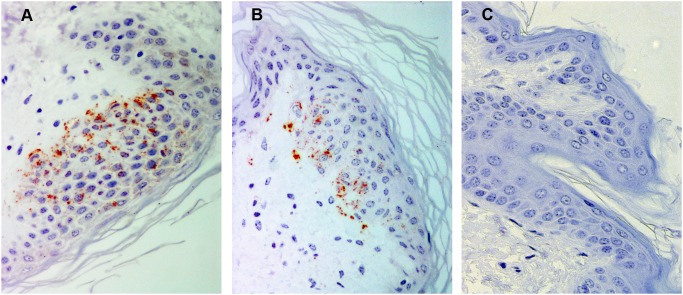
Immunohistochemical detection of PrP^Sc^ in skin. A–B) Skin from a naturally scrapie-infected sheep with PrP^Sc^ immunolabeling located mainly at the epidermis. PrP^Sc^ was detected using L42 (A; x40) and F89 (B; x40) PrP monoclonal antibodies. C) Skin from an uninfected control sheep in which no PrP^Sc^ immunolabeling is present (x40).

Accumulation of PrP^Sc^ in the skin was detected in only one sheep by both diagnostic techniques (No. 10; Table 2). By IDEXX EIA testing, this sheep exhibited a highly elevated OD value (2.56) similar to those obtained for spleen. By IHC, a granular labeling from the stratum basale to the stratum granulosum of the epidermis was observed and was likely associated with free nerve endings (Fig. 4A). Similar immunolabeling was observed with the F89 antibody (Fig. 4B). In 4 other sheep, PrP^Sc^ was detected in the skin solely by IHC (No. 2, 4, 7, 13). In these cases, PrP^Sc^ was associated with nerve fibres localised to the dermis as described in the experimental models [9]. Finally, a linear immunolabeling pattern localised to the basal side of the epithelium of the sweat glands was also observed. This pattern, whose specificity could not be confirmed, may be associated with the fibres that innervate these glands. No PrP^Sc^ immunolabelling was observed in skin of uninfected control sheep (Fig. 4C).

### Detection of PrP^sc^ in the Urinary Bladder

**Figure 5:**
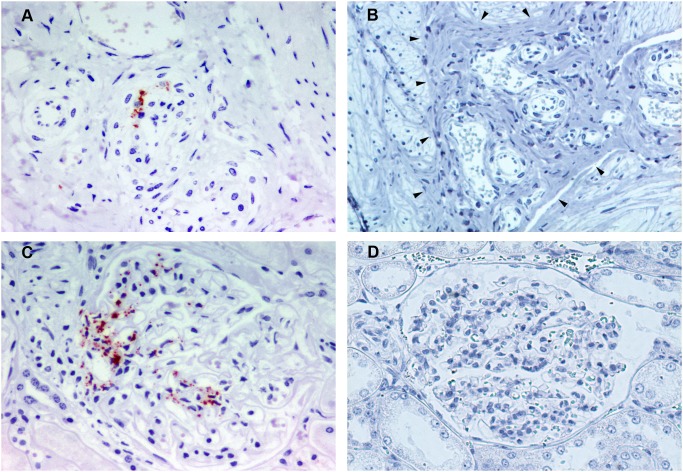
Immunohistochemical detection of PrP^Sc^ in urinary bladder and kidney using L42 PrP antibody. A) Urinary bladder muscularis from a naturally scrapie-infected sheep (x40). There is a weak PrP^Sc^ immunolabeling located in the connective tissue adjacent to the smooth muscle bundles, probably associated with vasculonervous elements. B) Urinary bladder muscularis from an uninfected control sheep in which no PrP^Sc^ immunolabeling is present (x20). The connective tissue (indicated by arrows head) with vasculonervous elements located between the smooth muscle can be clearly seen with low magnification. C) Kidney from a naturally scrapie-infected sheep. PrP^Sc^ immunolabeling is observed mainly at the vascular pole of a renal corpuscle (x40). D) A renal corpuscule of a kidney from an uninfected control sheep in which no PrP^Sc^ immunolabeling is present (x20).

In the urinary bladder, PrP^Sc^ was observed by IHC and IDEXX EIA in a single animal (No. 8; Table 2). A weak immunolabeling was detected in the connective tissue located among the smooth muscle fibres of the bladder wall (Fig. 5A). As with the previous organs, this pattern could be associated with the nerve fibres that innervate the bladder wall. Another sheep (No. 14) was positive only by IHC, although the OD value obtained by IDEXX EIA was elevated (0.16). In this sample, intracytoplasmic granular immunolabeling was found in the soma of a parasympathetic ganglia cell immersed in the bladder wall. No PrP^Sc^ immunolabelling was observed in urinary bladder of uninfected control sheep (Fig. 5B).

### Detection of PrP^sc^ in the Kidney

PrP^Sc^ deposits was detected in kidney samples from 6 infected sheep: 5 were positive by IHC only (No. 2, 4, 5, 10, 11; Table 2), and 1 was positive by IDEXX EIA only (No. 8). Seven samples displayed elevated OD values (range 0.10–0.21), although these values were below the cut-off point; 3 of these samples were positive by IHC. With one exception (No. 4), PrP^Sc^ immunolabeling was consistently found in the renal papillae interstice, as previously described [7]. In sheep No. 4, immunolabeling was observed at the vascular pole of a renal corpuscle of a juxtamedullary nephron in two consecutive sections (Fig. 5C). No PrP^Sc^ immunolabelling was observed in kidney of uninfected control sheep (Fig. 5D).

### Detection of PrP^sc^ in the Mammary Gland

**Figure 6:**
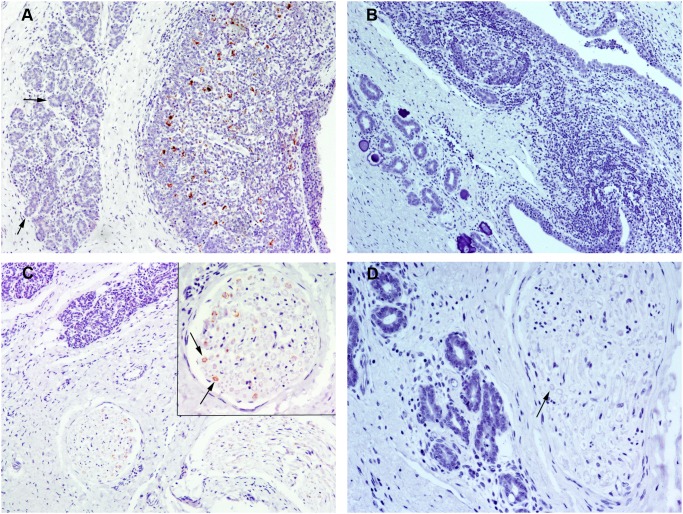
Immunohistochemical detection of PrP^Sc^ in mammary gland using L42 PrP antibody. A) Mammary gland from a sheep simultaneously infected by scrapie and by Maedi-visna infection causing a mastitis (x10). PrP^Sc^ immunolabeling is located within the lymphofollicular inflammatory lesions. In the mammary gland acini (arrows) next to the inflammatory lesions no PrP^Sc^ immunolabeling can be observed. B) Mammary gland from an uninfected control sheep with mastitis associated with Maedi-visna infection in which no PrP^Sc^ immunolabeling is present (x10). C) Mammary gland from a naturally scrapie-infected sheep (x10). PrP^Sc^ immunolabeling can be observed in the nerves immersed in the interlobular connective tissue (x10). A detailed image of a nerve fibers bundle in which a periaxonal PrP^Sc^ immunolabeling can be observed (arrows) is shown in the insert picture. D) Mammary gland from an uninfected control sheep in which no PrP^Sc^ immunolabeling is present (x20). Cross section of a nerve fiber is indicated by an arrow.

In the mammary gland, PrP^Sc^ was observed by IHC and IDEXX EIA in 2 sheep (No. 9, 12; Table 2), by IHC only in 2 sheep (No. 3, 7), and by IDEXX EIA only in 1 sheep (No. 10). The 2 animals that tested positive by both techniques, presented PrP^Sc^ deposition in lesions associated with Maedi-visna infection that consisted of lymphoid follicle hyperplasia (Fig. 6A). In these sheep, viral infection was confirmed by serum antibody detection using an ELISA test (ELITESTTM, Hyphen Biomed, Neuville sur Oise, France). Maedi-visna antibodies were also detected in a sheep with other lesions related to the disease (an interstitial inflammation; No. 14) and in 2 sheep with no lesions in the mammary gland (No. 11, 15). The 2 animals which were positive by IHC only (No. 3, 7) exhibited periaxonal immunolabeling in nerve fibres immersed in the interlobular connective tissue of the mammary glands (Fig. 6C). No PrP^Sc^ immunolabelling was observed in mammary gland of uninfected control sheep (Fig. 6B, D).

An epithelial immunolabeling pattern characterised by granular deposits at the basal side of the epithelium of the lactiferous ducts was observed in 3 sheep (No. 8, 10, 14; Fig. S1A). Although this immunolabeling pattern was no observed in mammary gland of uninfected control sheep (Fig. S1B), its specificity could not be demonstrated clearly because only one of these samples was positive by IDEXX EIA (No. 10). Thus, we classified this pattern as inconclusive by IHC.

### Detection of PrP^sc^ in the Lung

**Figure 7:**
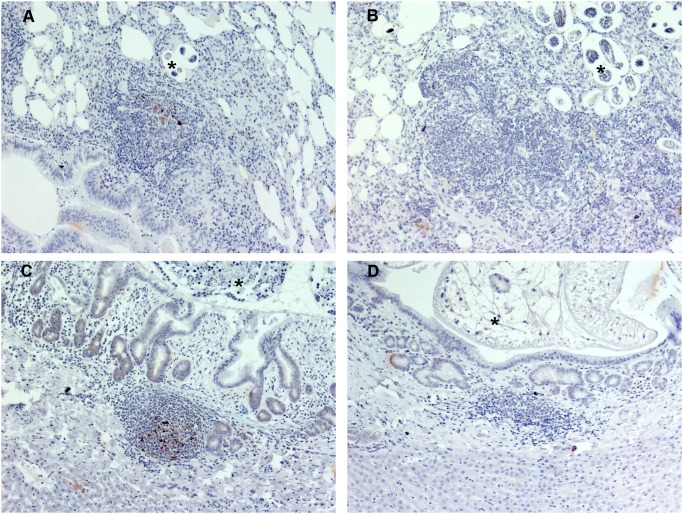
Immunohistochemical detection of PrP^Sc^ in lung and liver using L42 PrP antibody. A) Lung from a sheep infected by scrapie with a concomitant verminous pneumonia (x10). PrP^Sc^ immunolabeling is located within the inflammatory lesions related to the lungworm infection (x10). Parasites can be observed in the alveoli (asterisk). B) Lung from an uninfected control sheep with verminous pneumonia in which no PrP^Sc^ immunolabeling is present (x10). Parasites can be observed in the alveoli (asterik). C) Liver from a sheep infecetd by scrapie with a concomitant trematode infection (x10). PrP^Sc^ immunolabeling is associated with a lymphofollicular inflammatory site around a bile duct. In the bile duct lumen adult forms of parasites can be observed (asterisk). D) Liver from an uninfected scrapie sheep with trematode infection in which no PrP^Sc^ immunolabeling is present (x10). In the bile duct lumen, adult forms of parasites can be observed (asterisk).

The lung samples of 4 sheep were positive by IHC, but none of the samples were positive by IDEXX EIA. Moreover, all of the OD values were quite low (Table 2). In the positive samples, PrP^Sc^ accumulation was associated with chronic inflammatory lesions related to lungworm infection (No. 8, 13; Fig. 7A), Maedi-visna infection (No. 14) or both (No. 12). Maedi-visna infection was confirmed in sheep No. 12 and 14 by serum antibody detection as previously described. No PrP^Sc^ immunolabelling was observed in lung of uninfected control sheep (Fig. 7B). One sheep (No. 15) exhibited immunolabeling in the epithelium of the bronchioles, similar to that described in the mammary gland, which was classified as inconclusive by IHC (Fig. S1C, D).

### Detection of PrP^sc^ in the Liver

The liver samples of 3 sheep were positive by IHC only (No. 8, 10, 11; Table 2). In these animals, PrP^Sc^ was localised to lymphofollicular inflammatory sites around the bile ducts, in which adult forms of *Dicrocoelium* were occasionally observed in the lumen (Fig. 7C). No PrP^Sc^ immunolabelling was observed in liver of uninfected control sheep (Fig. 7D).

One sheep (No. 2) with no inflammatory lesions showed an immunolabeling pattern that has been previously associated with Kupffer cells [10]. Although this sample gave a relatively elevated OD value, it was below the cut-off point, and we considered it to be inconclusive by IHC (Fig. S2A, B).

## Discussion

The present study reveals that in ARQ/ARQ sheep with naturally acquired scrapie there is a wide dissemination of PrPSc in many organs outside the CNS and LRS. Although the tropism and distribution of PrPSc in sheep scrapie may be influenced by the ovine genotype [26], the majority of studies addressing the pathogenesis of naturally acquired scrapie have been performed using highly susceptible sheep (VRQ/VRQ). In the present study, 14 ARQ/ARQ sheep were included, all of them presenting scrapie clinical signs and a widespread PrPSc accumulation in the CNS. Considering only the organs that were positive by both of the PrPSc detection techniques in the same animal, 5 ARQ/ARQ sheep showed 2 or more positive peripheral organs. In these ARQ/ARQ sheep, in addition to being detected in the adrenal gland, PrPSc was detected by both IHC and IDEXX EIA in the pancreas and urinary bladder (No. 8), the pancreas (No. 4), the mammary gland (No. 9), the skin (No. 10) and the heart (No. 11). In sheep No. 8, 8 of the 11 peripheral organs studied were positive by at least one technique.

To our knowledge, this is the first report describing the presence of PrP^Sc^ in the pancreas, heart and urinary bladder in naturally acquired scrapie. Previously, infectivity in the pancreas in natural scrapie [26] and PrP^Sc^ accumulation in the pancreas and heart tissue in natural CWD [27], [28] has been described, although those studies reported a different localisation pattern from that observed in the present study. The results reported herein clearly show that PrP^Sc^ deposits in the pancreas are associated with the PNS; however, in natural and experimental CWD infections, PrP^Sc^ has been observed in the islets of Langerhans [27], [29]. It has been suggested that disturbances in endocrine homeostasis that have been described for several TSE, might be the result of targeting the prion infection to the peripheral and/or central endocrine system [30]. However, a relationship between the alteration of pancreatic endocrine function and PrP^Sc^ deposition in the islets of Langerhans has not been clearly shown [30]. Our results suggest that PrP^Sc^ deposition in the PNS (which innervates endocrine organs) may also participate in prion-induced endocrinopathies.

In the case of the heart, the high number of positive samples detected was not expected (8 out of 15 were positive by at least one technique), because the presence of PrP^Sc^ and its infectivity had not been previously reported in natural scrapie. In experimental and natural CWD, PrP^Sc^ deposits have been associated with cardiac muscle cells [28], [29]; however, our results show that immunolabeling of PrP^Sc^ is located between myocardial cells containing an extensive network of capillaries. Thus, PrP^Sc^ distribution in the heart is similar to the pattern described in a scrapie-infected transgenic mouse model that expresses a prion protein lacking the glycophosphatidylinositol membrane anchor. These mice deposit PrP^Sc^ in their hearts (primarily between myocytes and around capillaries), renal glomerulus, gut, and islets of Langerhans after scrapie infection, and their blood contained high levels of scrapie infectivity [31], [32]. In our kidney samples, the majority of PrP^Sc^ immunolabeling was observed in the renal papillae in accordance with previous studies [7]. However, in one case, granular immunolabeling at a renal corpuscle was observed. In addition to the rodent model, similar results in the kidney have been described in an FSE-infected cheetah [33].

In contrast, the number of PrP^Sc^ positive samples from urinary bladder detected was low. This result was expected (2 of 16 were positive by at least one technique) because infectivity has only been previously documented in an experimental model [34]. The single urinary bladder that was positive by both PrP^Sc^ detection techniques belonged to sheep No. 8, which exhibited the greatest PrP^Sc^ distribution throughout its body. This finding suggests that the urinary bladder is one of the last peripheral organs to be infected. As observed in the pancreas, the immunolabeling pattern observed in the urinary bladder was associated with the PNS components that innervate this organ. Additionally, PrP^Sc^ deposits were associated with PNS structures in the adrenal gland, mammary gland, skin and skeletal muscle.

In our study, the adrenal gland was the most affected peripheral organ because in all of the animals this tissue was at least positive by IHC. In sheep scrapie and other natural TSEs [27], [35], [36], [37], PrP^Sc^ deposition has been documented in the adrenal medulla associated with nerve fibres. In our samples, the majority of the PrP^Sc^ immunolabeling observed was also found in the medulla; however, it was associated with chromaffin cells, which are considered to be modified sympathetic postganglionic neurons. Recently, a similar pattern has been extensively described in natural and experimentally infected sheep [38]. In addition to the medulla, we also detected PrP^Sc^ deposits beneath the capsule associated with ganglion and nerve fibres, as well as in the suprarenal ganglion that surrounds the gland. To our knowledge, PrP^Sc^ immunolabeling in the adrenal cortex has only been described in a FSE-infected cheetah [33].

In the skeletal muscle (including the tongue) and the mammary gland, a periaxonal PrP^Sc^ immunolabeling pattern was observed in the small nerve fibre bundles immersed in the tissue. A similar immunolabeling pattern associated with Schwann cells has been described in peripheral nerves in experimentally induced scrapie infections [39], BSE in primates [40] and BSE in sheep [41]. In natural TSEs, PrP^Sc^ immunolabeling has been reported in muscle samples and was associated with intramuscular nerve fibres and muscle spindles in cases of scrapie [6] but was only associated with nerve fibres in human TSEs [42]. Consistent with Andreoletti *et al*. (2004), we observed PrP^Sc^ immunolabeling patterns that were associated with muscle spindles, but only in one sheep (No. 8), which had the greatest PrP^Sc^ distribution throughout its body. In the rest of the muscle samples, PrP^Sc^ immunolabeling was associated with intramuscular nerve fibres. We detected a higher number of positive muscle samples than Andreoletti *et al*. (2004), most likely because all of our sheep were in an advanced clinical phase of the disease.

Regarding the skin, the presence of PrP^Sc^ has been demonstrated by WB in natural scrapie infection and by WB, PET blot and IHC in a rodent model, showing that PrP^Sc^ is always associated with small nerve fibres within the dermis [9]. Although we have also observed this immunolabeling pattern, we describe for the first time the presence of PrP^Sc^ within the epidermis, which was most likely associated with free nerve endings. Our findings confirms the skin as a potential reservoir of prions that could play a role in the spread of scrapie in the field, as it has been suggested previously [9] [43].

We observed an epithelial immunolabeling pattern associated with sweat glands, lacteal ducts and bronchioles. In some cases, the topographical location of the immunolabeling (on the basal side of the epithelium) suggests that this pattern could be associated with nerve endings. In the case of the mammary gland and sweat glands, this location could also suggest the immunolabeling of myoepithelial cells; however, previous reports have shown that these cells do not express PrP^c^ in the ovine mammary gland [44], [45]. Although natural scrapie infections exhibiting an intracellular immunolabeling pattern associated with the epithelial cells of the salivary glands have been previously reported [46], we classified our samples as inconclusive by IHC. Only one sample exhibiting this exclusive epithelial immunolabeling pattern was also positive by IDEXX EIA (No. 10; mammary gland); however, because the samples subjected to IHC and IDEXX EIA were not the same, we cannot dismiss the possibility that this positive result could be due to the presence of positive nerve fibre bundles within the tissue.

A large body of evidence indicates that scrapie infection ascends from the gut into the thoracic spinal cord and to the medulla oblongata along the splanchnic and vagus nerves, respectively. From these initial sites, infection propagates cranially and caudally within the CNS, which subsequently seems to spread centrifugally to the sensory ganglia of the vagus and splanchnic circuits (for review see [17]). PrP^Sc^ centrifugal transport from the brain to further peripheral tissues along peripheral nerves has been demonstrated mainly in animal models and through intracerebral inoculation [6], [9], [47], [48]. Our results are in agreement with these experimental models and suggest that there is a marked spread of PrP^Sc^ from the CNS to other tissues, mainly throughout the PNS in natural scrapie infections. All of the animals included in the present study showed a widespread distribution of PrP^Sc^ within the CNS. In peripheral organs, we localised PrP^Sc^ to structures associated with sympathetic efferent nerves (i.e., chromaffin cells in the adrenal gland), parasympathetic efferent nerves (i.e., neurons immersed in the pancreatic parenchyma or the urinary bladder walls) and sensory nerves (i.e., epidermis). PrP^Sc^ immunolabeling observed in nerves within skeletal muscle could be associated with sensory or somatic motor nerve fibers.

In our study, the pelvic visceral organs (urinary bladder and reproductive organs) were the least affected. Unlike the abdominal and thoracic viscera, the cell bodies of the preganglionic parasympathetic neurons that innervate the pelvic viscera are located in the sacral spinal cord. In addition, the sensory ganglia are also associated with this spinal cord segment. Because the sacral segment is the most caudal region of the spinal cord, the slow progression of infection from the initial site of CNS invasion at the thoracic level to the sacral level, and from there to the pelvic organs, could explain why these are some of the last peripheral organs to be infected. The rate of spread of the scrapie agent within the nervous system could also explain why the greatest PrP^Sc^ dissemination is observed in the ARQ/ARQ animals. In the present study, only 2 VRQ/VRQ sheep were included, but neither of them exhibited the highest number of positive peripheral organs. It has been shown that there is a large genetic influence on the incubation period of scrapie in sheep [49] and it has been suggested that polymorphisms in the PrP gene may alter PrP^Sc^ accumulation kinetics rather than accumulation sites or cellular specificity [50]. The progression of PrP^Sc^ through the nervous system could be slower in ARQ/ARQ sheep than in VRQ/VRQ sheep. Thus, ARQ/ARQ sheep will develop the disease after a longer incubation period, and they have more time for PrP^Sc^ to spread throughout the PNS. Consistent with this, one of the oldest animals included in the present study (No. 8; ARQ/ARQ genotype) showed the greatest PrP^Sc^ distribution throughout its body. Previously, it has been reported that a prolonged incubation period together with a marked LRS involvement increases the probability of PrP^Sc^ accumulation in sheep kidneys, although in this case, a hematogenous spreading mechanism was proposed [7], [10]. In contrast, our results suggest that PrP^Sc^ dissemination in the organism does not appear to be influenced by the degree of LRS involvement because 2 sheep exhibited several non-lymphoid positive organs, and all of the lymphoid organs that were studied were negative.

Although the PNS seems to be the main mechanism of PrP^Sc^ dissemination in a host, inflammation or the presence of prions in the blood may lead to a more widespread and pronounced accumulation of PrP^Sc^ deposits in the body [11]. In addition to PNS structures, we detected PrP^Sc^ immunolabeling associated with chronic inflammatory lesions. In sheep with naturally acquired scrapie, PrP^Sc^ deposition has been described previously in abomasum, mammary gland and lung tissue associated with viral and/or parasitic infections [13], [15], [51]. However, to our knowledge, this is the first description of the presence of PrP^Sc^ in liver tissue associated with chronic inflammatory lesions in naturally scrapie-infected sheep. Recently, PrP^Sc^ deposition has been detected in liver tissue with no inflammatory lesions from naturally infected VRQ/VRQ sheep and was found to be associated with Kupffer cells [10]. Interestingly, we observed a similar pattern of immunolabeling in only one sheep which exhibited a VRQ/VRQ genotype; nevertheless, we could not confirm the presence of PrP^Sc^ by IDEXX EIA. It has been proposed that accumulation of PrP^Sc^ in Kupffer cells and renal papillae results from direct exposure to prions in the blood [7], [10]. The hematogenous spread could also explain the PrP^Sc^ deposits that we observed in the vascular pole of a renal corpuscle. However, the sympathetic nerve fibres that reach that zone of the corpuscle and innervate the juxtaglomerular cells might also explain this renal PrP^Sc^ accumulation. Likewise, the origin of the PrP^Sc^ accumulation that we have observed in the heart remains unclear, and further investigations are needed to clarify the PrP^Sc^ location at a cellular level.

Overall, the results from the present study showed large variation in peripheral spread of PrP^Sc^ among sheep. Although all of the infected animals included in the study presented a widespread PrP^Sc^ accumulation in the CNS, they did not show a similar PrP^Sc^ dissemination through the PNS. The heterogeneous distribution of PrP^Sc^-containing nerve fibres within the peripheral organs in conjunction with the small amounts observed could explain the variation between animals. This variation has also been observed in the skeletal muscle [6], kidney [7], liver [10] and skin [9] of sheep infected with scrapie. It should be highlighted that only 2 pieces of tissue were collected from fairly big organs, one for IHC and one for EIA test. Sampling multiple sites within organs and using more sensitive techniques would most likely result in a higher number of peripheral positive organs in each animal, but the study is not intended to be representative in nature. In addition, the results from the IHC and IDEXX EIA analyses showed a poor correlation between both techniques, with a higher number of positive samples observed via IHC in comparison with the IDEXX EIA. This IDEXX EIA is a commercial immunoassay for PrP^Sc^ detection in nervous or lymphoid tissues. In the present study, all samples were analysed and interpreted according to the manufacturer’s cut-off criterion. In some previous studies that have used commercial ELISA tests for PrP^Sc^ detection in non-nervous or non-lymphoid tissues, the cut-off value has been recalculated as a function of the type of tissue analysed (as 3 standard deviations above the mean of the negative controls [10], [41]). The cut off values determined in these cases were lower than those set by the manufacturers (i.e., 0.059 for liver samples [10]). Equally, we calculated the cut off for liver, pancreas and urinary bladder from 10 negative sheep (as 4 standard deviations above the mean) and obtained values of 0.069, 0.040 and 0.063, respectively. If these cut off values were used, all of the samples that were positive by IHC only, would be confirmed by IDEXX EIA; however, we would also have 1 positive sample only by IDEXX EIA in the case of liver tissue, 7 in the pancreas and 2 in the urinary bladder. Therefore, although it is possible that by applying the manufacturer’s cut-off criterion we would obtain a lower IDEXX EIA sensitivity, a more in-depth study is needed to adapt the cut off value for each individual tissue.

Together with previous reports, the present study indicates that scrapie infection may spread through the PNS to most, if not all, parts of the body. The PrP^Sc^ presence in non-nervous organs does not appear to have any additional pathological role but demonstrates that some of these organs are potentially infectious (which has not been considered thus far [21]) and generates new insights into horizontal transmission. In addition, our observations regarding naturally acquired scrapie may provide indirect information about the spread of the agent in related TSEs such as vCJD and CWD.

## Supporting Information

Figure S1 **Immunolabeling classified as inconclusive by immunohistochemiestry in mammary gland and lung.** A) Mammary gland from a naturally scrapie-infected sheep (x40). A granular immunolabeling at the basal side of the epithelium of a lactiferous duct can be observed. B) A lactiferous duct of a mammary gland from an uninfected control sheep in which no immunolabeling is present (x40). C) Lung from a naturally scrapie-infected sheep (x40). Immunolabeling in the epithelium of a bronchiole can be observed. D) A bronchiole of a lung from an uninfected control sheep in which no immunolabeling is present (x40). The specificity of the epithelial immunolabeling pattern observed in (A) and (C) could not be confirmed by IDEXX EIA.
(TIF)

Figure S2 **Immunolabeling classified as inconclusive by immunohistochemiestry in liver.** A) Liver from a naturally scrapie-infected sheep (x63). An immunolabeling probably related to a Kupffer cell (arrow) was observed. This immunolabeling was detected only in one sample and its specificity could not be demonstrated because this sample was negative by IDEXX EIA. B) Liver from an uninfected control sheep in which no immunolabeling is present (x63). Kupffer cells are indicated by arrows.
(TIF)

Table S1 **Details of control sheep used in the study.** From each animal the identification number, age, flock of origin and genotype are included.
(DOCX)

Table S2 **Individual sheep results of PrP^Sc^ detection by IHC/IDEXX EIA.** The OD value obtained by IDEXX EIA is indicated for samples that were assessed by this technique.
(DOCX)
