# Supplementary figures and images for: Distribution of Peripheral PrPSc in Sheep with Naturally Acquired Scrapie
Source: PLoS One. 2014 May 14;9(5):e97768. doi: 10.1371/journal.pone.0097768 (PMC4020850; doi:10.1371/journal.pone.0097768)

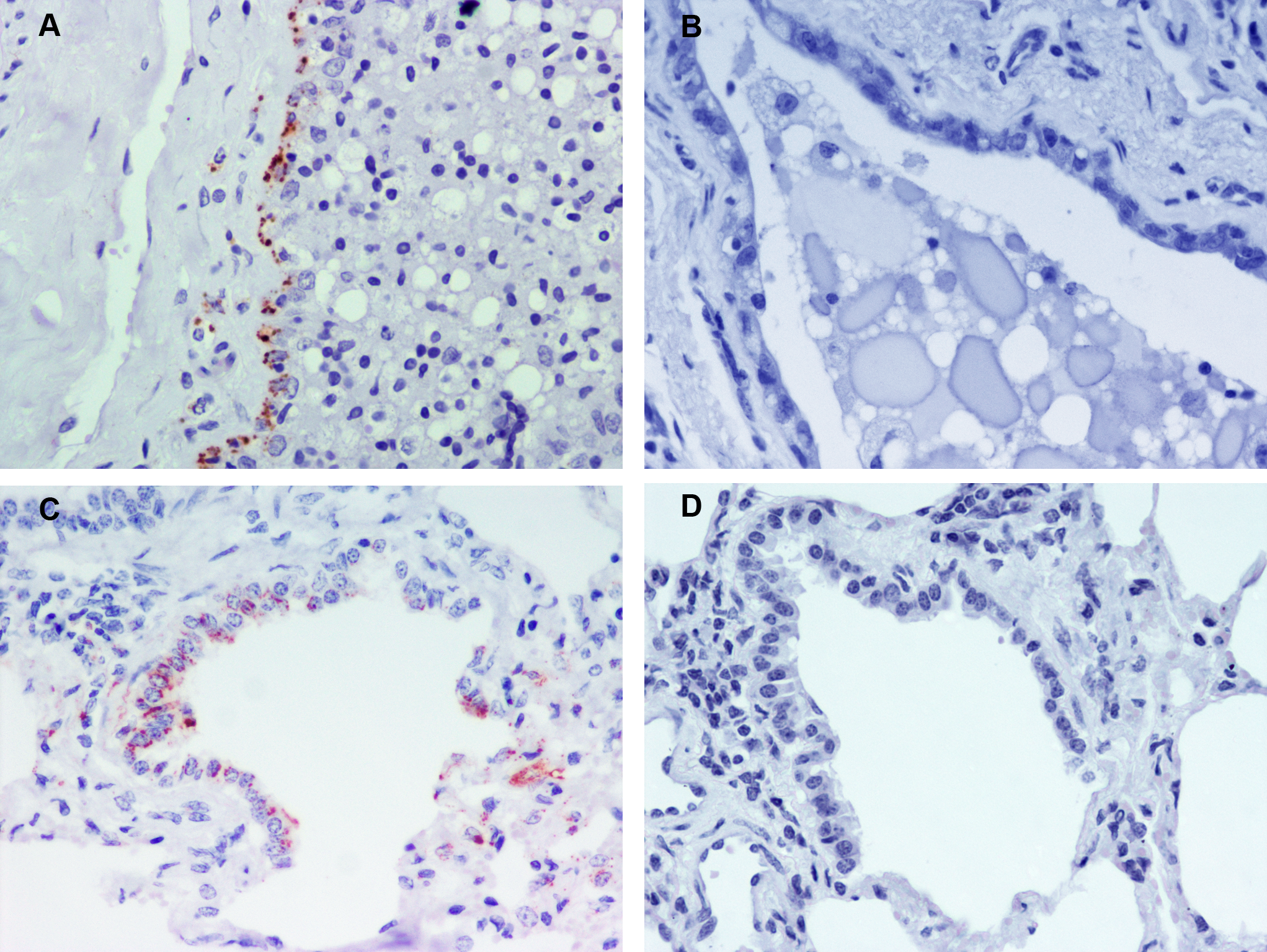

Supplement: Figure S1 — Immunolabeling classified as inconclusive by immunohistochemiestry in mammary gland and lung. A) Mammary gland from a naturally scrapie-infected sheep (x40). A granular immunolabeling at the basal side of the epithelium of a lactiferous duct can be observed. B) A lactiferous duct of a mammary gland from an uninfected control sheep in which no immunolabeling is present (x40). C) Lung from a naturally scrapie-infected sheep (x40). Immunolabeling in the epithelium of a bronchiole can be observed. D) A bronchiole of a lung from an uninfected control sheep in which no immunolabeling is present (x40). The specificity of the epithelial immunolabeling pattern observed in (A) and (C) could not be confirmed by IDEXX EIA. (TIF) [file pone.0097768.s001.tif]

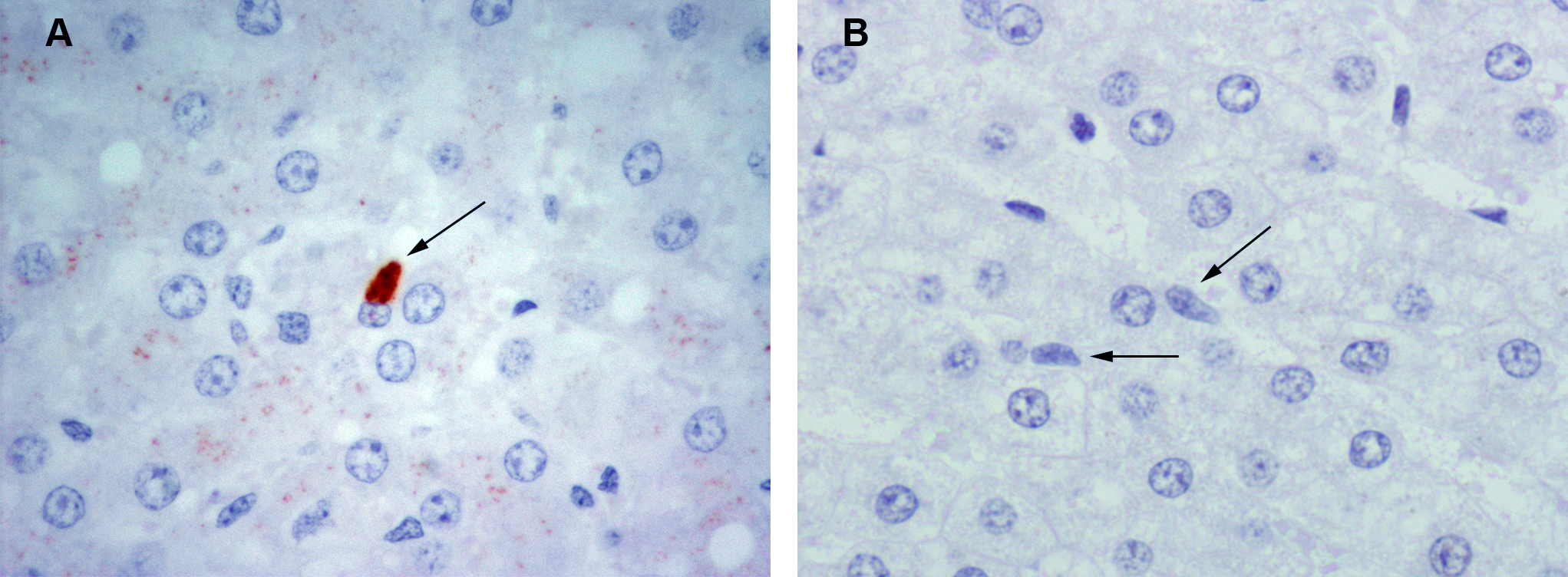

Supplement: Figure S2 — Immunolabeling classified as inconclusive by immunohistochemiestry in liver. A) Liver from a naturally scrapie-infected sheep (x63). An immunolabeling probably related to a Kupffer cell (arrow) was observed. This immunolabeling was detected only in one sample and its specificity could not be demonstrated because this sample was negative by IDEXX EIA. B) Liver from an uninfected control sheep in which no immunolabeling is present (x63). Kupffer cells are indicated by arrows. (TIF) [file pone.0097768.s002.tif]
