# Supplementary material for: Distribution of Peripheral PrPSc in Sheep with Naturally Acquired Scrapie
Source: PLoS One. 2014 May 14;9(5):e97768. doi: 10.1371/journal.pone.0097768 (PMC4020850; doi:10.1371/journal.pone.0097768)
Supplement: Table S1 — Details of control sheep used in the study. From each animal the identification number, age, flock of origin and genotype are included. (DOCX) [file pone.0097768.s003.docx]

**Table S1.** Details of control sheep used in the study. From each animal the identification number, age, flock of origin and genotype are included.

| **Animal No.** | **Age^1^ (years)** | **Outbreak** | **Genotype** |
| --- | --- | --- | --- |
|  |  |  |  |
| 1C | >6 | A | VRQ/VRQ |
| 2C | >6 | B | VRQ/ARQ |
| 3C | >6 | C | VRQ/ARQ |
| 4C | >6 | D | ARQ/ARQ |
| 5C | >6 | E | ARQ/ARQ |
| 6C | 4 | F | ARQ/ARQ |
| 7C | 5 | G | ARQ/ARQ |
| 8C | >6 | H | ARQ/ARQ |
| 9C | 5 | C | ARR/ARQ |
| 10C | 4 | D | ARR/ARQ |

**^1^** Age was determined by the dental status of the animals.
