# Supplementary material for: Distribution of Peripheral PrPSc in Sheep with Naturally Acquired Scrapie
Source: PLoS One. 2014 May 14;9(5):e97768. doi: 10.1371/journal.pone.0097768 (PMC4020850; doi:10.1371/journal.pone.0097768)
Supplement: Table S2 — Individual sheep results of PrPSc detection by IHC/IDEXX EIA. The OD value obtained by IDEXX EIA is indicated for samples that were assessed by this technique. (DOCX) [file pone.0097768.s004.docx]

**Table S2.** Individual sheep results of PrP^Sc^ detection by IHC / IDEXX EIA. The OD value obtained by IDEXX EIA is indicated for samples that were assessed by this technique.

|  | **Animal No.** | | | | | | | | | |
| --- | --- | --- | --- | --- | --- | --- | --- | --- | --- | --- |
|  | **1C** | **2C** | **3C** | **4C** | **5C** | **6C** | **7C** | **8C** | **9C** | **10C** |
| **Brainstem** | -/- | -/- | -/- | -/- | -/- | -/- | -/- | -/- | -/- | -/- |
|  | 0.026 | 0.033 | 0.024 | 0.032 | 0.022 | 0.018 | 0.021 | 0.021 | 0.03 | 0.029 |
| **Spleen** | -/- | -/- | -/- | -/- | -/- | -/- | -/- | -/- | -/- | -/- |
|  | 0.034 | 0.049 | 0.026 | 0.022 | 0.025 | 0.021 | 0.022 | 0.015 | 0.045 | 0.035 |
| **Adrenal gl.** | -/NT | -/NT | -/NT | -/NT | -/NT | -/NT | -/NT | -/NT | -/NT | -/NT |
| **Heart** | -/NT | -/NT | -/NT | -/NT | -/NT | -/NT | -/NT | -/NT | -/NT | -/NT |
| **Tongue** | -/NT | -/NT | -/NT | -/NT | -/NT | -/NT | -/NT | -/NT | -/NT | -/NT |
| **Sk. Muscle** | -/NT | -/NT | -/NT | -/NT | -/NT | -/NT | -/NT | -/NT | -/NT | -/NT |
| **Pancreas** | -/- | -/- | -/- | -/- | -/- | -/- | -/- | -/- | -/- | -/- |
|  | 0.034 | 0.027 | 0.019 | 0.022 | 0.023 | 0.026 | 0.024 | 0.025 | 0.02 | 0.029 |
| **Urinary bl.** | -/- | -/- | -/- | -/- | -/- | -/- | -/- | -/- | -/- | -/- |
|  | 0.02 | 0.031 | 0.03 | 0.02 | 0.023 | 0.001 | 0.02 | 0.035 | 0.03 | 0.03 |
| **Skin** | -/NT | -/NT | -/NT | -/NT | -/NT | -/NT | -/NT | -/NT | -/NT | -/NT |
| **Kidney** | -/NT | -/NT | -/NT | -/NT | -/NT | -/NT | -/NT | -/NT | -/NT | -/NT |
| **Mamm. Gl.** | -/NT | -/NT | -/NT | -/NT | -/NT | -/NT | -/NT | -/NT | -/NT | -/NT |
| **Lung** | -/NT | -/NT | -/NT | -/NT | -/NT | -/NT | -/NT | -/NT | -/NT | -/NT |
| **Liver** | -/- | -/- | -/- | -/- | -/- | -/- | -/- | -/- | -/- | -/- |
|  | 0.024 | 0.029 | 0.034 | 0.049 | 0.026 | 0.028 | 0.024 | 0.024 | 0.041 | 0.045 |

NT, no tested.
